# Implications of Post-recanalization Perfusion Deficit After Acute Ischemic Stroke: a Scoping Review of Clinical and Preclinical Imaging Studies

**DOI:** 10.1007/s12975-022-01120-6

**Published:** 2023-01-19

**Authors:** Noa van der Knaap, Bart A. A. Franx, Charles B. L. M. Majoie, Aad van der Lugt, Rick M. Dijkhuizen, on behalf of the CONTRAST consortium

**Affiliations:** 1 Biomedical MR Imaging and Spectroscopy Group, Center for Image Sciences, University Medical Center Utrecht and Utrecht University, Utrecht, The Netherlands; 2 Department of Radiology and Nuclear Medicine, Amsterdam University Medical Center, Location University of Amsterdam, Amsterdam, The Netherlands; 3 Department of Radiology & Nuclear Medicine, Erasmus MC University Medical Center Rotterdam, Rotterdam, The Netherlands

**Keywords:** Acute ischemic stroke, Reperfusion, Recanalization, Perfusion deficit, Hyperperfusion, Hypoperfusion

## Abstract

The goal of reperfusion therapy for acute ischemic stroke (AIS) is to restore cerebral blood flow through recanalization of the occluded vessel. Unfortunately, successful recanalization does not always result in favorable clinical outcome. Post-recanalization perfusion deficits (PRPDs), constituted by cerebral hypo- or hyperperfusion, may contribute to lagging patient recovery rates, but its clinical significance remains unclear. This scoping review provides an overview of clinical and preclinical findings on post-ischemic reperfusion, aiming to elucidate the pattern and consequences of PRPD from a translational perspective. The MEDLINE database was searched for quantitative clinical and preclinical studies of AIS reporting PRPD based on cerebral circulation parameters acquired by translational tomographic imaging methods. PRPD and stroke outcome were mapped on a charting table, creating an overview of PRPD after AIS. Twenty-two clinical and twenty-two preclinical studies were included. Post-recanalization hypoperfusion is rarely reported in clinical studies (4/22) but unequivocally associated with detrimental outcome. Post-recanalization hyperperfusion is more commonly reported (18/22 clinical studies) and may be associated with positive or negative outcome. PRPD has been replicated in animal studies, offering mechanistic insights into causes and consequences of PRPD and allowing delineation of possible courses of PRPD. Complex relationships exist between PRPD and stroke outcome. Diversity in methods and lack of standardized definitions in reperfusion studies complicate the characterization of reperfusion patterns. Recommendations are made to advance the understanding of PRPD mechanisms and to further disentangle the relation between PRPD and disease outcome.

**Supplementary Information:**

The online version contains supplementary material available at 10.1007/s12975-022-01120-6.

## Introduction

Reperfusion therapy after acute ischemic stroke (AIS) aims to recanalize the occluded artery and preserve still viable penumbral tissue [1]. Current FDA-approved reperfusion therapy options include intravenous thrombolysis (IVT) with recombinant tissue plasminogen activator (rtPA) and endovascular thrombectomy (EVT). Yet, despite these advancements, the outlook remains grim for a considerable number of AIS patients.

Although recanalization strategies have improved over the years, more than a third of successfully recanalized AIS patients show no clinical improvement, otherwise known as “futile recanalization” [2], an effect that cannot be completely explained by initial ischemic severity or final infarct size [3, 4]. The cause of these disappointing patient recovery rates is an area of intense research wherein neuroimaging plays a major role. Magnetic resonance imaging (MRI) and computed tomography (CT), integral to diagnosis and treatment decision-making, also allow longitudinal monitoring of the course of reperfusion and infarct progression in AIS patients. Cerebral perfusion can be measured with perfusion CT, which is readily available in clinical practice but requires iodinated contrast agents and exposure to radiation. Alternatively, two perfusion MRI techniques are available, dynamic susceptibility contrast-enhanced MRI (DSC-MRI) and arterial spin labeling (ASL), of which the latter does not require use of a contrast agent and is therefore entirely non-invasive. These techniques are used to diagnose AIS patients but have also identified post-recanalization perfusion deficits (PRPDs) [5–7], which are hypothesized to play a significant role in disease outcome [8, 9]. In this context, PRPD refers to an abnormal cerebral perfusion state after recanalization, often expressed as the level of cerebral blood flow (CBF), which can be decreased (hypoperfusion) or increased (hyperperfusion) relative to perfusion in normal brain tissue or a certain cut-off value.

Barring the most obvious culprits, such as recanalization failure or re-occlusion, a well-known hypothesis for post-recanalization hypoperfusion is incomplete microvascular reperfusion (IMR), which posits that the microvascular bed remains obstructed after treatment and therefore hampers tissue recovery [10]. On the other hand, post-recanalization hyperperfusion, in earlier literature recognized as “luxury perfusion” and used to describe a state of perfusion exceeding metabolic demands of tissue [11], may also be related to lagging clinical recovery. This excessive cerebral perfusion has previously been associated with complications in stroke [12] and carotid artery stenting [13]. Conversely, hyperperfusion has also been regarded as the hallmark of successful recanalization [14, 15].

Historically, animal models of ischemic stroke have provided valuable insights into stroke pathophysiology where human data was unavailable. For example, “neuroprotection” and “penumbra” are concepts that originated from experimental stroke research [16]. Models of ischemia–reperfusion have been (longitudinally) studied in imaging studies for years, often with the same imaging techniques available for clinical diagnostics. The breakthrough of EVT renewed interest in futile recanalization and some of the classic AIS models in the experimental stroke field [17]; therefore, this body of literature merits re-evaluation as it could generate new insights and hypotheses. However, a coherent overview of observed PRPDs is still lacking.

The main objective of this scoping review is to map the present clinical and preclinical evidence pertaining to cerebral PRPD after (experimental) recanalization treatment. Importantly, we aim to build a bridge between clinical and preclinical reports to identify possible causes and consequences of PRPD, such that new hypotheses may be generated to advance the field of AIS management.

## Methods

### Study Design

The scoping review method was selected because we wished to summarize evidence of in vivo PRPD obtained by means of medical imaging, as well as to identify research gaps in the existing literature [18]. The scoping review method is useful for summarizing literature that addresses research questions using different instruments and definitions to describe the same phenomenon (such as ASL and DSC-MRI to measure CBF), increasing the breadth of the available literature and thus rendering our inquiry intractable for systematic review.

### Protocol

The rapid scoping review protocol was drafted and findings are reported using the checklist provided in the Preferred Reporting Items for Systematic Reviews and Meta-analysis extension for Scoping Reviews (PRISMA-ScR) [18]. The protocol was revised internally by the research team and is outlined below.

### Eligibility Criteria

Three major inclusion criteria were defined prior to literature search. Peer-reviewed journal papers needed to (1) concern focal ischemic stroke exclusively, (2) employ medical imaging tools able to measure and spatially resolve cerebral perfusion after (spontaneous) recanalization (e.g., MRI or CT), and (3) describe post-recanalization cerebral (re)perfusion. Only quantitative and mixed-method studies, which included human patients or animals as study sample, were considered. Only papers written in English were selected for inclusion, with no limit on publishing period. Case studies were excluded. Experimental stroke studies were also excluded if non-medical imaging techniques were used that cannot be used for brain imaging in the clinical setting, such as laser Doppler flowmetry, which would hamper our attempts to translate findings to humans.

### Information Sources and Search Strategy

The literature search was performed on the MEDLINE database, last accessed on July 1st, 2022. Two separate search formulae were set up for clinical and preclinical studies. The main keywords used in both formulae included cerebral ischemia or stroke or brain infarction; magnetic resonance imaging or computed tomography or perfusion imaging; and reperfusion or recanalization. For the full search formulae, see Table 1. References of the selected articles were screened for additional relevant articles.

**Table 1:** Search formulae for clinical and preclinical studies

Clinical search formula	Preclinical search formula
((ischemic stroke[mh]) OR (cerebral ischemia) OR (brain infarction[mh]) OR (brain ischemia[mh])) AND ((magnetic resonance imaging[mh]) OR (computed tomography[mh]) OR (positron emission tomography[mh])) AND ((perfusion imaging[mh]) OR (“cerebral blood flow”) OR (“cerebral blood volume”) OR ((ASL) OR (arterial spin labeling)) OR (dynamic susceptibility*)) AND ((reperfusion) OR (recanalization) OR (mechanical thrombectomy) OR (thrombolyis) OR (tPA)) AND ((hypoperfusion) OR (hyperperfusion) OR (perfusion deficit[tw]) OR (perfusion)) NOT ((cardi*) OR (renal) OR (heart) OR (“drug effects”[sh]) OR (“drug therapy”[sh]) OR (review[Filter])) AND (humans[Filter])	((ischemic stroke[mh]) OR (cerebral ischemia) OR (brain infarction[mh]) OR (brain ischemia[mh])) AND ((magnetic resonance imaging[mh]) OR (computed tomography[mh]) OR (positron emission tomography[mh])) AND ((perfusion imaging[mh]) OR (“cerebral blood flow”) OR (“cerebral blood volume”) OR (ASL) OR (arterial spin labeling) OR (dynamic susceptibility*)) AND ((reperfusion) OR (recanalization)) AND ((hypoperfusion) OR (hyperperfusion) OR (“luxury perfusion”) OR (perfusion deficit[tw]) OR (perfusion)) NOT ((cardi*) OR (renal) OR (heart) OR (“drug effects”[sh]) OR (“drug therapy”[sh]) OR (review[Filter])) AND (animal[Filter])

### Study Selection

**Fig. 1:**
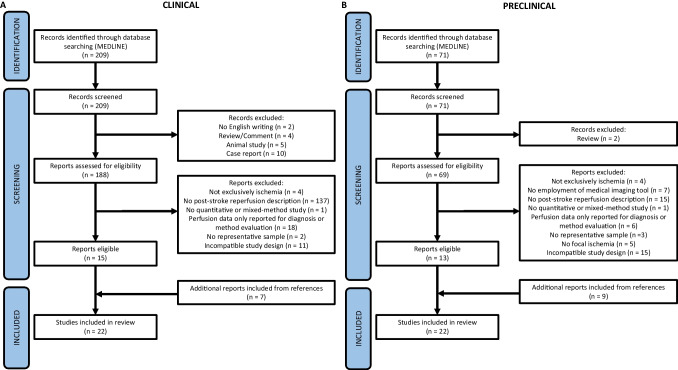
PRISMA flowchart for study selection process. Study selection flowcharts of clinical (**A**) and preclinical (**B**) studies

Literature search results were manually screened and further narrowed down based on title and abstract by N.v.d.K. and B.A.A.F., who also screened the full-text articles after final selection had been made. Disagreements on study selection and data extraction were resolved by consensus and discussion with experienced reviewers (A.v.d.L, C.B.L.M.M, and R.M.D.) if needed. The study selection process is presented in Fig. 1 for clinical (Fig. 1A) and preclinical studies (Fig. 1B).

### Data Abstraction Process

We anticipated that key variables to our research question, cerebral perfusion and disease outcome, would be assessed and reported using a wide variety of methods available in both clinical and preclinical settings. We therefore set out to abstract these variables from quantitative to qualitative status. For example, cerebral perfusion after ischemic stroke can be measured over several time points using ASL or CT perfusion (CTP) techniques and quantified as deficient using relative or absolute perfusion parameters. We used a predefined abstraction form to express post-stroke perfusion as normoperfusion (no change from baseline), hyperperfusion (perfusion overshoot), or hypoperfusion (perfusion insufficiency), regardless of the technique employed. As clinical definitions for hypo- or hyperperfusion are often lacking or controversial, our classification was guided by statistical tests and interpretation of the data by the authors. Disease outcome can be expressed using functional outcomes or imaging markers in the clinic, while ex vivo tissue analyses are conventional in the preclinical setting. Here, we also sought to convert the continuous or discrete variable to a qualitative statement. Then, in order to map a possible timeline of PRPD, salient data (patient groups, experimental groups, or even tissue voxel clusters) from each clinical and preclinical study were categorized depending on disease outcome and tabulated relative to stroke onset time. Particularly for clinical papers, the process of mapping PRPD to the correct relative time category (bin) was highly dependent on detailed reporting of stroke onset (last-known well time), treatment onset, and binning strategy where variability in patient imaging examination times is concerned. Most studies related follow-up imaging to stroke onset, but other events such as treatment onset were also used. In this case, we reasoned based on other reported time parameters and the maximum treatment window of a particular therapy and, to the best of our ability, estimated to which time bin after stroke onset a particular result belonged. The abstraction process was completed when N.v.d.K and B.A.A.F. reached consensus, and the result was quality checked by the remaining experienced reviewers (A.v.d.L, C.B.L.M.M, and R.M.D.). For two studies [19, 20], the data abstraction process could not be completed; these are discussed below but not included in Supplementary Table 1.

### Risk of Bias Assessment

Risk of bias assessment was not conducted as is consistent with (rapid) scoping reviews on health-related topics [21].

### Data Synthesis

Results of the abstraction process are presented in Supplementary Tables 1 and 2. Below, the reader is provided with a disquisition about the study selection, akin to a narrative analysis, to contextualize our analysis of the possible relationship between PRPD and disease outcome.

## Results

### Literature Search

The clinical and preclinical search formulas resulted in 209 and 71 hits, respectively. Preliminary screening resulted in fifteen and thirteen unique titles for the clinical and preclinical search, respectively. Seven additional clinical titles and nine additional preclinical titles were identified through screening references and expert advice (Fig. 1). Study-specific methods and time-bound reperfusion data are summarized in Supplementary Tables 1 and 2, for clinical and preclinical studies respectively. For the sake of brevity, some studies have only been abstracted without further discussion due to lack of mechanistic implications.

### PRPD in a Clinical Setting

#### Persisting Post-recanalization Hypoperfusion

##### Definition

Although post-recanalization hypoperfusion is not formally defined, workable heuristics may be extracted from studies of the ischemic threshold. Seminal animal experiments have demonstrated that there are time-dependent relationships between CBF thresholds and tissue viability [22]. In order to help identify the infarct core and penumbra, this concept has been translated to aid clinical decision-making. Assuming baseline CBF of 50 ml/100 g/min (gray matter), a drop to roughly 35–40% of baseline incapacitates electrical activity and may closely represent the dominant notion of penumbra, while a drop below 20–24% triggers cytotoxic edema [23]. Three recent studies used thresholding methods on CBF maps, identifying regional hypoperfusion as a 15% [24], 20% [25], or 40% [26] drop below a reference CBF value, obtained from a contralateral region-of-interest representing a fair control. Although it can be argued that CBF is the most relevant parameter to identify hypoperfused tissue, preference is often given to time-to-maximum (Tmax) maps, which represent the time when the amplitude of the voxel-wise deconvolved residue function is maximal [6]. Presumably, maps of non-physiological timing parameters like Tmax are preferred because these parameters can be more stable and easier to calculate than CBF (and by extension, MTT), exhibiting less variability than CBF (which varies by tissue type, i.e., gray vs. white matter), and the perfusion lesion on Tmax is hyperintense and possibly easier to distinguish [6, 27]. Multiple studies have used a conventional Tmax delay ≥ 6 s to delineate malignant hypoperfusion [28–32], as this parameter setting has shown to be a good predictor of final infarct volume [33–35]. Another method that has been used to study hypoperfusion evaluates the hypoperfusion intensity ratio (HIR), which is defined as the ratio between the volume of tissue with severe hypoperfusion (e.g., Tmax ≥ 10 s) and the volume of tissue with any hypoperfusion (e.g., Tmax ≥ 6 s) [20, 36]. High HIR is thought to reflect more malignant hypoperfusion and poor collaterals. It should be noted that the exact definition of HIR is study-specific, as other Tmax cutoffs have been used [37], which can limit generalizability of results. Finally, the HIR can be an ambiguous representation of hypoperfusion abnormality after AIS; i.e., in cases where the tissue volume with Tmax > 6 s reduces over time while the volume of Tmax > 10 s remains constant, the HIR will increase (to a maximum of 1.0), while the total volume of hypoperfusion is decreasing.

##### Clinical Significance of Hypoperfusion

To assess the severity of post-recanalization hypoperfusion in relation to disease outcome, Olivot et al. [20] retrospectively evaluated HIRs from AIS patients, including 99 patients who received EVT within 12 h after stroke onset. Perfusion deficit progression from pre- to post-recanalization was determined by comparison of HIR 12 h after recanalization therapy versus baseline, and recanalization was graded on angiographic images. In general, patients without reperfusion, thus persisting hypoperfusion deficit, displayed HIR increase, while patients who reperfused early exhibited significant HIR decrease. These data suggest that hypoperfusion can worsen over time in patients with incomplete or no reperfusion; however, there was no significant relationship between HIR increase and clinical outcome.

Rubiera et al. [32] evaluated 151 EVT-treated AIS patients with CTP within 30 min after EVT and showed hypoperfusion in 52.9% of the patients. Post-recanalization hypoperfusion volume was significantly associated with the degree of recanalization, as measured by the modified thrombolysis in cerebral ischemia (mTICI) scale, with larger hypoperfusion volumes in patients in whom complete recanalization was not achieved. Overall, patients with larger hypoperfusion volumes had less favorable outcome 3 months after stroke.

An association between regional hypoperfusion after complete recanalization, thought to represent no-reflow, with unfavorable outcome has been established [24]. By visually grading regions of hyperperfusion, verified by a CBF threshold of ≤ 15% of the contralateral value, no-reflow was observed in 33 out of 130 (25.3%) IVT- and EVT-treated patients. Local no-reflow was associated with hemorrhagic transformation (HT), greater infarct growth, and worse mRS scores on 90-day follow-up. In contrast, Ter Schiphorst et al. [26], using a CBF threshold of ≤ 40% of the contralateral value, observed no-reflow in one out of 33 (3%) EVT-treated patients with complete recanalization, who had favorable clinical outcome. This suggests that no-reflow after complete recanalization in man may be rare and might not contribute to futile recanalization.

Recently, Rosso et al. [25] set out to identify and quantify unique hypo- and hyperperfusion profiles with ASL in and around the ischemic core in 226 patients, 24 h after receiving IVT. In 40.9% of patients, hypoperfusion was found in the lesion. Hypoperfusion in and around the lesion was found in 12.4% of patients and associated with a poor 90-day mRS score (details on hyperperfusion-related findings can be found in the “Hyperperfusion and Favorable Outcome” section). Notably, the rate of complete recanalization, as graded on angiographic images, was at least 40.7% in patients exhibiting hypoperfusion in the diffusion-weighted imaging lesion.

#### Post-recanalization Hyperperfusion

##### Definition

Hyperperfusion, which can be described by an increase of CBF of > 100% of baseline [38], sits at the other end of the reperfusion abnormality spectrum. Although formal definitions are lacking, some studies have applied CBF ≥ 130% of control (contralateral) values as hyperperfusion threshold [39, 40], whereas other studies relied on qualitative assessment [41, 42]. Tmax maps may theoretically be used to delineate areas of hyperperfusion, but threshold values have not been published. Based on our own unpublished data, we speculate MRI-derived Tmax is not preferred because a short Tmax may be missed if the TR of a pulse sequence is too long (e.g., when using spin-echo sequences). Further, in our experience, the maximum of the tissue concentration curve in hyperperfused areas arrives only a little earlier compared to normoperfused areas. There is relatively more variation in magnitude of the deconvolved residue function (i.e., CBF), which leads to more fine-grained detail of hyperperfusion on CBF maps.

##### Hyperperfusion and Favorable Outcome

Based on positron emission tomography (PET) data, Marchal et al. [9] were one of the first to report that early hyperperfusion (within 24 h) after spontaneous recanalization in untreated AIS patients was associated with favorable tissue outcome. Replicating this finding several years later, the same group additionally observed that early hyperperfused areas did not overlap with areas that eventually infarcted [14]. After 1 month, CBF had normalized in previously hyperperfused areas, and neurological scores, measured after 2 months, had improved in all included patients (*n* = 10). In a study with 119 patients, of which 100 IVT-treated patients, Bhaskar et al. observed perilesional hyperperfusion at 24 h after symptom onset in 42 patients with deep subcortical infarcts or cortical MCA territory infarcts, of which 37 received treatment [43]. Perilesional hyperperfusion was associated with successful reperfusion (as graded by mTICI ≥ 2b), penumbral salvage, and favorable clinical trajectory.

Where Marchal et al. [14] and Bhaskar et al. [43] mainly described perilesional hyperperfusion, Crisi et al. [44] reported hyperperfusion closely around and within the lesion in 22 out of 47 untreated AIS patients, measured with ASL 24-to-36 h after stroke onset. Patients with hyperperfusion had lower National Institutes of Health Stroke Scale (NIHSS) scores at admission. The authors concluded that intra-lesional hyperperfusion may have a protective role by preventing ischemia from spreading. However, the effect of hyperperfusion on lesion evolution was not addressed in the analysis.

Viallon et al. [45] assessed a group of 41 AIS patients of whom the majority received antiplatelet treatment and seven received IVT treatment. At an average post-stroke time of 48 h, hyperperfusion within and around the lesion was observed in twelve patients (of whom three received IVT treatment) who all had good outcome at 3-month follow-up. Recanalization rates were not graded. In all these patients, the acute hyperperfusion volume was larger than the final infarct volume. Hyperperfused areas, not part of the infarct core, were associated with complete tissue recovery. The authors suggested that hyperperfusion was therefore unlikely to be the cause of tissue necrosis. Patients with either hyperperfusion (29.3%) or no perfusion deficit (34.1%) had better outcome than patients with persisting hypoperfusion (36.6%).

Rosso et al. [25] found similar relationships between outcome and regional hyperperfusion in patients during MRI examination 24 h after IVT (hypoperfusion-related findings are discussed in the “Clinical Significance of Hypoperfusion” section). Perfusion abnormality profiles existing of a hyperperfused ischemic core, combined with either hyper- (34.5%) or normoperfusion (19.5%) in the surrounding tissue, were associated with complete recanalization grades and improved clinical outcome, which were absent in patients with persisting hypoperfusion in and around the ischemic core (12.4%). Patients with hypoperfused lesions but hyperperfused surrounding tissues (17.7%) exhibited an intermediate clinical trajectory between persisting hypoperfusion or other varying hyperperfusion profiles.

In a prospective study by Bivard et al. [46], 89 AIS patients were recruited in an imaging study consisting of baseline CTP within 6 h of symptom onset and follow-up MRI examination, including ASL and DSC-PWI. Of these patients, 41 were treated with intravenous thrombolysis. Complete recanalization was graded by a previously published angiography-guided recanalization scale. Perfusion status was dichotomized into “major reperfusion” and “no reperfusion” based on a reduction of the perfusion lesion from baseline to 24-h follow-up of > 80% or < 20%, respectively. Hyperperfusion was observed in 43 patients, all of whom achieved complete recanalization. Hyperperfusion on ASL scans was more likely in patients who received thrombolysis. There was a high correlation between major reperfusion and hyperperfusion as seen on ASL scans in regions that were ischemic during acute imaging: both groups showed similar improvements in penumbral salvage and functional outcome. However, compared to persisting hypoperfusion, only patients with regional hyperperfusion exhibited improved NIHSS scores, reduced infarct growth, and increased penumbral salvage. Less infarct growth and increased penumbral salvage were also observed in patients with hyperperfusion. Similarly, in a follow-up study by Bivard et al. [39], patients with hyperperfusion had better clinical outcome at 3-month follow-up than patients without hyperperfusion. Here, major reperfusion was used as inclusion criterion, and hyperperfusion was observed around the infarct at 24 h post-treatment in 24 out of 77 patients.

In a retrospective study of 54 EVT-treated patients, 36 patients showed hyperperfusion in the infarcted area on ASL-based scans at 4 days (median) after stroke onset, which was related to successful recanalization and associated with an improvement in NIHSS at 24 h. Hyperperfusion was, independently from successful recanalization, also associated with good functional outcome at 90 days post-stroke [40]. Potreck et al. [41] visually graded hyperperfusion using DSC-MRI at 24 h after successful EVT in a prospective study of 38 patients who achieved complete recanalization (mTICI ≥ 2b). Here, perfusion patterns within the infarcted region were assessed, leading to three distinct patterns: predominant hyperperfusion, predominant hypoperfusion, or normoperfusion. Hyperperfusion was measured in seventeen patients, normoperfusion (no perfusion deficit) in thirteen patients, and persisting hypoperfusion in seven patients. Patients with either normoperfusion or hyperperfusion were more likely to have a favorable outcome on the mRS at 90 day follow-up than patients with persisting hypoperfusion.

##### Hyperperfusion and Neutral or Unfavorable Outcome

Not all studies in which hyperperfusion was detected after recanalization reported a positive effect on clinical outcome. Kidwell et al. [47] prospectively observed hyperperfusion at 7 days in six out of eleven AIS patients who received intra-arterial thrombolysis with or without IVT and demonstrated clinical improvement, but clinical improvement was not significantly different between these patients and patients without hyperperfusion. In this study, hyperperfusion was observed both on early (5 h after treatment) and late (7 days after treatment) MRI, where hyperperfusion was larger in volume and more severe in intensity on MRI after 7 days. Hyperperfusion, which progressively increased between 5 h and 7 days after IVT, was evident in areas that ultimately became infarcted but not in the entire area of final infarction. These areas were more severely ischemic before treatment, as indicated by lower apparent diffusion coefficient (ADC) values and a greater decrease in CBF (hypoperfusion) than areas that did not progress to hyperperfusion. The authors therefore reasoned that hyperperfusion was mainly caused by impaired autoregulation rather than a normal response to hypermetaNot all studies in which hyperperfusion was detected after recanalization reportedbolism.

Several other studies linked hyperperfusion to worse prognoses. Shimonaga et al. [42] retrospectively studied data from 27 patients treated with EVT and found correlations between visually graded post-recanalization hyperperfusion and prolonged disturbance of consciousness, poor functional outcome, and HT. Recanalization rates were not reported. Yu et al. [48] attempted to retrospectively distinguish HT grades in treated and untreated patients based on early and late ASL readouts of cerebral perfusion. HT occurred more frequently in patients with late hyperperfusion (> 12 h after stroke onset), with a positive relationship between HT grade and time to hyperperfusion, whereas NIHSS scores were not significantly different between the early and late hyperperfusion groups. Recanalization rates were not graded. These results suggest that hyperperfusion is particularly harmful when it occurs at later stages.

In a retrospective study of EVT-treated patients, both hyper- and hypoperfusion were associated with HT [49]. In another retrospective study in treated and untreated patients, HT was asymptomatic, yet more prevalent in tissue that reperfused and that displayed more severe initial perfusion deficit at baseline [19]. Also, it has been reported that parenchymal hematomas, the most severe manifestions of HT, associate more often with hypoperfusion than hyperperfusion in and around the ischemic core after IVT at 24 h post-stroke [25].

### PRPD in a Preclinical Setting

AIS is a complex and heterogeneous disease, and cerebral perfusion is difficult to longitudinally assess in patients as it is not standard of care. Therefore, animal models have been employed to obtain detailed knowledge on post-stroke hemodynamics, which can be translated to clinical situations. Although there are many different approaches to experimentally induce ischemic stroke, the intraluminal filament middle cerebral artery occlusion (MCAO) model has been used most often [17]. Filament retraction, where retraction delay represents a modifiable experimental variable, reinstates flow through the MCA, allowing assessment of the effects of recanalization, including the course of reperfusion and tissue changes. It has been suggested that this model resembles mechanical thrombectomy in a clinical setting [17]. The studies discussed below have used the MCAO model, unless otherwise specified. Important to note is that time to recanalization has a strong relationship with ischemic tissue injury and can have different species-dependent effects on experimental outcomes. In rodents, for example, the effects of a relatively short duration of MCAO (30 min) in mice resemble effects of longer MCAO durations in rats (60–120 min) [50].

A wide array of (imaging) techniques is available to monitor the course of perfusion changes in animal stroke models, yet studies with medical imaging modalities like MRI, CT, PET, and single photon emission compute tomography (SPECT) allow most straightforward translation through the use of similar protocols as in the clinic [51].

#### Preclinical Findings of PRPD

##### Hypoperfusion in AIS Models

Hyperacute post-recanalization hypoperfusion has been detected in a longitudinal MRI study of AIS in rats by Bardutzky et al. [52]. CBF was monitored with MRI during ischemia and after in-bore recanalization in rats, 35 or 95 min after MCAO. During reperfusion, monitored up to 180 min after occlusion, post-ischemic tissue remained in a hypoperfused state, although tissue that ultimately recovered, in general, reached higher CBF values than tissue that ultimately became infarcted. At 24 h after occlusion in the 35-min MCAO group, hyperperfusion was measured in the infarct area.

Burrows et al. [53] showed that post-recanalization hypoperfusion in mice may be a delayed response. Reperfusion was assessed by measuring the cerebral oxyhemoglobin (HbO_2_) concentration with SPECT. After a significant decrease in HbO_2_ concentration during ischemia, an early (8–15 min) increase in perfusion was observed after filament removal. However, in a later reperfusion phase (345–360 min), a second episode of significant HbO_2_ decrease was observed in the post-ischemic area, with only major vessels retaining HbO_2_ increase. This delayed reperfusion deficit (hypoperfusion) was accompanied by early inflammatory processes, microglia activation, blood–brain barrier (BBB) breakdown, and neuronal loss.

Similarly, Van Dorsten et al. [54] showed with ASL that recanalization after 60-min MCAO in mice induced slow and incomplete reperfusion. In contrast, the same group [55] performed a similar experiment in rats and observed immediate reperfusion after recanalization with return of CBF to normal (pre-ischemic) levels. These findings suggest that the early course of reperfusion after experimental MCAO may be species-dependent.

Early hypoperfusion after recanalization may be related to experimental factors resembling comorbidity like hyperglycemia or hypertension. In a DSC-MRI study, hyperglycemic rats displayed 37% lower cerebral blood volume (CBV) (as compared to contralesional control values) in the post-ischemic area following 2-h MCAO, up to several hours after recanalization, while normoglycemic rats showed normalized cerebral perfusion levels after recanalization [56]. Tissue injury, as measured with diffusion-weighted MRI, overlapped with the area of CBV hypoperfusion. In another MRI study, CBF, measured with ASL, did not recover in spontaneously hypertensive rats after 1-h MCAO and led to progression of infarction [57]. This contrasted with normotensive rats that displayed rapid CBF normalization and smaller lesion volumes after transient MCAO (tMCAO). It is important to note, however, that although tissue and perfusion status may appear normal on MRI after recanalization, post-ischemic tissue may contain irreversibly damaged neurons, and neurological deficits may persist [58].

##### Hyperperfusion in AIS Models

As described above, post-recanalization reperfusion levels may vary as a function of time. In PET studies by Martín et al. [59, 60], immediate normalization of CBF was measured after tMCAO in rats (1 h after recanalization), followed by a phase of hypoperfusion on day 1. After day 4 and onto day 7, a second increase in CBF above contralateral baseline occurred, reflecting hyperperfusion. CBF returned to normal levels after day 14 [60]. Immunohistochemistry showed an increase in microvessel density from day 2 onwards, which the authors suggested may have been responsible for hyperperfusion.

Increased vascular density in the post-stroke rat brain has also been observed by Lin et al. [61]. Hyperperfusion in the ipsilateral hemisphere was first measured with MRI at day 1 and peaked at day 7 after recanalization and coincided with increased vascular density.

In cats, PET revealed hyperperfusion immediately after recanalization in the 30- and 60-min MCAO groups [62]. Where CBF returned to normal levels from 30 min onward in the 30-min occlusion group, hyperperfusion lasted longer and affected a larger tissue volume in the 60-min group. Notably, all cats in the 30-min occlusion group survived, while six out of eleven cats in the 60-min occlusion group died. Hyperperfusion after recanalization in surviving cats was of shorter duration and to lesser degree, and final infarct size was smaller. These findings suggest that a longer state of hypoperfusion during occlusion results in a longer period of hyperperfusion after recanalization, leading to worse (tissue) outcome.

Also in rats, hyperperfusion after recanalization has been linked to poorer tissue outcome. In an MRI study by Shen et al. [63], no early hyperperfusion was observed in the first 3 h after recanalization, but late hyperperfusion occurred after 48 h in rats that underwent 30-min (all animals) or 60-min MCAO (some animals) but not in animals with 90-min MCAO. Hyperperfusion was associated with poor tissue outcome. A negative CBF response to a CO_2_-challenge was present after 48 h, suggesting disrupted autoregulation in hyperperfused tissue. Hyperperfusion was resolved at day 7.

An MRI study by Wegener et al. [64] confirmed that hyperperfusion after recanalization reduces vasoreactivity and that longer duration of hyperperfusion may lead to larger infarct size. Based on ASL data, they dichotomized three different patterns of hyperperfusion in rats after 60-min MCAO: (1) hyperperfusion on day 4, increasing toward day 14; (2) hyperperfusion on day 1, peaking at day 4 and remitted toward day 14; and (3) a maximum state of hyperperfusion on day 1, stabilizing or decreasing thereafter. The first two patterns were observed in animals with cortical and subcortical damage, while the third pattern was seen in animals with only subcortical infarctions. Neurological deficiency scores were collected at day 14 but these did not correlate significantly with the amount of hyperperfused voxels.

Post-ischemic hyperperfusion has also been measured in a rat embolic stroke model, in which intraluminal injection of an autologous blood clot was followed by IVT after 1 h [65]. Perfusion MRI revealed regional hyperperfusion at 2 and 48 h after stroke onset, indicating that post-ischemic hyperperfusion can also develop under conditions of more gradual recanalization after pharmacological thrombolysis.

##### PRPD and Reperfusion Injury

There are several studies that indicate reperfusion in and of itself contributes to cellular damage, commonly referred to as reperfusion injury, and that PRPD may be an aspect or epiphenomenon of this process. Secondary tissue injury has been reported after normalization of CBF in rats after 30-min MCAO [66, 67], although this did not hamper functional recovery [66]. In a study in cynomolgus monkeys, 3-h tMCAO led to immediate post-recanalization hyperperfusion followed by hypoperfusion in cortical areas in which necrotic brain damage developed [68]. The extent of brain damage was greater after transient MCAO compared to permanent MCAO, suggesting that reperfusion, through post-recanalization hyper- or hypoperfusion, significantly contributes to post-ischemic tissue injury. Similarly, in cats [69] and rats [70], cellular damage was identified in post-ischemic areas with hyperperfusion at 2 to 4 days after recanalization.

Interestingly, post-recanalization BBB leakage, regarded as a manifestation of reperfusion injury, has been associated with PRPD on several occasions. In an MRI study comparing permanent, 1-h and 3-h tMCAO in rats, 3-h tMCAO was found to lead to early CBV hyperperfusion followed by normalization [71]. Disruption of the BBB, as measured with dynamic contrast-enhanced MRI (DCE-MRI), was identified immediately in this group after recanalization, but both BBB leakage and CBV hyperperfusion were absent in animals subjected to 1-h tMCAO. The authors postulated that hyperperfusion was attributable to compensatory dilation of perilesional collaterals and exacerbated BBB leakage. Finally, another MRI study (including ASL and DCE-MRI) of 1-h tMCAO in rats demonstrated normalized perfusion and an intact BBB immediately after recanalization but later co-localized areas of hyperperfusion and BBB permeability after 2 days [72].

## Discussion

This scoping review aimed to provide a methodical overview of the current evidence of PRPD after AIS, gathered from clinical and preclinical studies with medical imaging techniques. Through this method, we have depicted possible courses of PRPD, associated with favorable or unfavorable disease outcome (Supplementary Tables 1 and 2). Factors that may explain causes and consequences of post-recanalization perfusion deficits have been identified, which we discuss below for hypoperfusion and hyperperfusion. Lastly, we describe remaining knowledge gaps and propose methodological improvements that could enhance study translatability and generalizability and ultimately improve our understanding of this important phenomenon.

### Post-recanalization Hypoperfusion: Prognostic Value, Possible Causes, and Consequences

Reperfusion therapy is grounded in the idea that restoration of CBF is the best conceivable treatment against cerebral ischemia. In that sense, persisting hypoperfusion after recanalization is a probable candidate to explain lagging patient recovery rates. Accumulating evidence indicates that post-recanalization hypoperfusion is detrimental to stroke outcome [20, 24, 25, 32, 41]. However, the underlying causes are unclear and require scientific attention. Aside from treatment failure, arterial reocclusion may be a prime suspect. It has been estimated that arterial reocclusion occurs in up to a third of patients treated with IVT and may occur as late as 2 h after treatment [2]. Therefore, arterial reocclusion may be a causative mediator of hypoperfusion in the context of thrombolytic reperfusion therapy. In AIS patients undergoing EVT, reocclusion is less common but associated with poor clinical outcome [73, 74]. Thus, conceivably, treatment strategy can be a risk factor for post-recanalization hypoperfusion.

Experimental stroke studies highlight several factors that may contribute to post-recanalization hypoperfusion in the absence of arterial reocclusion. Bardutzky et al. [52] have suggested that persistent acute hypoperfusion is exhibited by ischemic tissue destined for infarction and that the most important predictor for this tissue fate was occlusion duration. Other studies have shown that comorbidities related to poor vascular health may promote a state of post-recanalization hypoperfusion, as shown in experiments with hyperglycemic [56] and spontaneously hypertensive rats [57]. These data are clinically relevant as they may explain disease outcomes in patients that present with vascular comorbidity.

In preclinical settings, choice of animal species and occlusion times also affect PRPD. Post-recanalization hypoperfusion is more often documented in mouse models of AIS than in rat models, illustrated in Supplementary Table 2. In the studies discussed in this review, persisting hypoperfusion in mice was observed by Van Dorsten et al. [54] and Burrows et al. [53], yet conversely, studies in rats predominantly reported post-recanalization normo- or hyperperfusion. Such inter-species differences may be at least partially explained by the dissimilar impact occlusion time has in different animals: e.g., 60-min tMCAO in mice is considered more severe compared to the impact of 60-min tMCAO in rats [54]. Moreover, species difference in the vascular anatomy can be an intermediating variable to consider with respect to tMCAO outcome. A recent study highlighted differences in vascular anatomy and infarction volume between various rodent species subjected to experimental AIS, demonstrating that mice tend to have relatively fewer collateral vessels and increased lesion sizes [75].

Persisting obstruction of the microvascular bed after successful recanalization (i.e., IMR) has been well-described in certain animal models based on microscopy measurements [10, 76, 77], yet this phenomenon remains poorly characterized in man. An attempt has been made to clinically identify IMR on CT angiography [78], where “capillary blush” induced by the contrast agent distal to the occlusion site was considered an indicator of capillary status and found predictive of unsatisfactory outcome. A recent MRI study observed regions of no-reflow in 33 out of 130 patients with successful recanalization after EVT, which were associated with HT, greater infarct growth and poor outcome [24]. This study can be juxtaposed however by Ter Schiphorst et al. [26], who reported only one case of overt hypoperfusion (with good clinical outcome) out of 33 patients with complete recanalization. Thus, clinical evidence for IMR and/or no-reflow is limited. Yet, the work by Burrows et al. [53] suggests that post-recanalization hypoperfusion can indicate a global state of no-reflow and manifest as a delayed response after recanalization. Taken together, it remains challenging in both the clinical and preclinical setting to identify post-recanalization hypoperfusion as IMR using conventional whole-brain tomographic imaging methods because they lack sensitivity to microvascular flow compared to high-resolution microscopy techniques. Experimental studies combining clinically translatable imaging and high-resolution microscopy could shed more light on the occurrence and detectability of IMR.

### Post-recanalization Hyperperfusion: Prognostic Value, Possible Causes, and Consequences

Results from our data abstraction indicate hyperperfusion may occur more frequently compared to hypoperfusion. In addition, although the implications of hyperperfusion are feared and universally considered malignant under certain conditions, such as after carotid artery stenting [13], our assessment shows no clear-cut relationship with outcome in AIS, both clinically (Supplementary Table 1) and preclinically (Supplementary Table 2).

Unfortunately, clinical mechanistic studies of hyperperfusion are lacking. To the best of our knowledge, only one clinical study has attempted to explain post-stroke hyperperfusion in relation to outcome by elucidating the metabolic profile of hyperperfused penumbra using magnetic resonance spectroscopy imaging [39]. Here, hyperperfused penumbral tissue scanned 24 h after treatment showed a distinct metabolic profile compared to non-hyperperfused tissue and healthy tissue in healthy control subjects. Peri-infarct glutamate concentrations were elevated in patients with hyperperfusion compared to healthy controls, whereas these concentrations were lower in patients without hyperperfusion compared to controls. As speculated by the authors, the elevated glutamate concentrations in reperfused peri-infarct tissue may be related to increased cellular activity. Additionally, the concentration of *N*-Acetylaspartate, considered to be a marker of neuronal density and mitochondrial metabolism, was significantly increased in hyperperfused tissue as compared to non-hyperperfused patients and controls.

#### Hyperperfusion, a Predictor of Good Outcome

Multiple clinical studies have reported an association between post-ischemic hyperperfusion and favorable clinical outcome [9, 14, 25, 39–41, 43–46]. Several studies observed hyperperfusion outside of the final infarct [14, 25, 43, 45], which could be suggestive of hyperperfusion being a marker of penumbral salvage. Hyperperfusion within the lesion, on the other hand, may prevent infarct expansion [44]. These studies demonstrate that there may be no distinguishable spatial hyperperfusion pattern that could be a predictor for a good outcome. In animal AIS models, hyperperfusion has primarily been found to overlap with the cerebral ischemic lesion [63, 64, 69, 70] and has been associated with various pathological mechanisms that are inextricably linked to other aspects of AIS pathology. Notably, we found only one study that included a functional outcome measure, reporting that hyperperfusion did not have a clear effect on functional outcome [64].

An important factor that may be involved in the beneficial effect hyperperfusion imparts to functional outcome is the condition of collaterals or arterial health in general. Bhaskar et al. [43] showed that perilesional hyperperfusion was associated with a good collateral vessels status at the time of hospital admission, thus before patients received treatment. Other clinical studies have shown that baseline collateral status affects infarct growth and penumbral salvage [79] and clinical outcome post-recanalization [36, 80]. Additionally, the condition of pre-treatment collateral flow could influence the success of recanalization after treatment [81], both after IVT and EVT [36]. Preclinical studies testing causal hypotheses about the relation between regional hyperperfusion and collateral status in AIS after recanalization are lacking.

#### Hyperperfusion, a Predictor of Detrimental Outcome

HT is a feared complication in the context of AIS and has been linked to post-recanalization hyperperfusion [42, 48, 49, 82]. At present, mechanisms underlying HT are incompletely understood, and a causal relationship between HT and hyperperfusion is yet to be established. It has been speculated that tissue severely damaged by ischemia is more at risk for HT after reperfusion [39, 48] and that a link between HT and hyperperfusion may be explained through impaired autoregulation [48]. Further, HT has, similar to hyperperfusion, been associated with other overlapping mechanisms of AIS pathophysiology such as persisting hypoperfusion [25], BBB disruption [83, 84], and poor collaterals [81]. Together, these data illustrate the complexity of the disease process and multifaceted mechanisms underlying hyperperfusion and HT.

Preclinical studies provide some support for abovementioned clinical observations. Impaired autoregulation in rodents has been measured in hyperperfused areas [63, 64], and it has been linked to prolonged hyperperfusion after tMCAO [65]. Studies that reported occurrence of HT only observed it in animals with late and prolonged hyperperfusion [70] or prolonged hyperperfusion in general [62]. Additionally, HT was associated with increased post-stroke injury in rats with comorbidities, such as old age and hypertension [85]. Taken together, many hypotheses of directional links between hyperperfusion, impaired autoregulation, and HT remain. There is an unmet demand for mechanistic explanations as to how patients develop HT and how it may be prevented.

#### Hyperperfusion and Reperfusion Injury

Reperfusion injury is a looming and unwanted phenomenon of reperfusion therapy, where the cerebral post-ischemic tissue fails to benefit from recanalization but rather deteriorates as a result. Studies have shown that hyperperfusion may be related or lead to (secondary) BBB disruption [69, 72], cell damage [68–70], and HT [48]. However, preclinical studies that have associated hyperperfusion with tissue injury after recanalization emphasize that it is difficult to distinguish damage attributable to reperfusion injury from initial ischemic damage [69, 70].

Post-ischemic reperfusion injury continues to pose significant challenges to interpretation of neuroprotection trials and AIS management, with robust evidence from the laboratory, whereas clinical prevalence is still contested [86]. Reperfusion injury constitutes a highly complex cascade of biochemical processes that can antagonize tissue recovery, but it still lacks complete descriptions of cause-and-effect for major pathways. In addition, various definitions of reperfusion injury exist; HT has been considered a clinical manifestation of reperfusion injury, for example, yet the underlying etiology of the syndromes may differ [87]. Currently, the underlying mechanisms of reperfusion injury remain to be elucidated and so are the possible roles that PRPD assumes in this phenomenon.

#### Prognostic Value of Hyperperfusion

The body of literature presented in this review depicts a complex landscape of evidence attributing both detrimental and beneficial effects to hyperperfusion after AIS. Conceivably, if there is a long-term prognostic value to hyperperfusion, it may depend on temporal and spatial conditions. Firstly, prognostic value could be a function of time, both in terms of occurrence and duration. Several early reports have already suggested hyperperfusion shortly after reperfusion therapy may be a harmless [15] or beneficial phenomenon [14], supported by various subsequent retrospective trials [25, 39–41, 43–46]. Mechanistic evidence supporting this notion could be found clinically in metabolic profiles of hyperperfused penumbral tissue at 24 h [39]. Preclinical studies primarily reported detrimental outcomes for hyperperfused tissue but did so in the absence of functional outcome. One preclinical study that did report such measures showed occurrence of hyperperfusion may be neutral [64]. Conversely, late hyperperfusion may signify increased risk of HT [48], although some clinical studies have associated late hyperperfusion to favorable outcome as well (Supplementary Table 1). Presently, the interpretation and definition of *late* hyperperfusion are impeded by clinical evidence consisting of only a single imaging time point, a non-trivial consideration given the highly dynamic nature of PRPD [59, 60]. Longitudinal data from animal studies indicate an association of late and *prolonged* hyperperfusion with cellular damage [70, 72], impaired vasoreactivity [63, 64], larger infarct size [64], poor condition of blood vessels [63], and HT [62, 70].

Second, the spatial location of hyperperfusion may explain its prognostic value. Hyperperfusion in perilesional tissue has been shown to reduce lesion growth and/or improve outcome [14, 25, 43, 45], also corroborated by evidence of favorable metabolic profiles [39]. However, in preclinical studies, hyperperfusion often overlapped with infarcted tissue [47, 69, 70] and secondary tissue injury [63, 72]. Together, these results indicate that hyperperfusion may occur in both penumbral and irreversibly damaged tissue, where it may not always be a recovery mechanism. In that sense, spatial distributions of hyperperfusion alone, without the use of imaging techniques to differentiate potentially salvageable from perished tissue, may perform poorly as outcome predictors.

### Outstanding Questions, Challenges, and Future Perspectives

Our interpretation presented above is limited by several caveats. Cerebral perfusion is often implicitly treated as an isolated system, while its components belong to the overarching and intricate cardiovascular system. As mentioned, malignant PRPD may be linked to loss of cerebral autoregulation, which in itself is shown to correlate with unfavorable clinical outcome [88]. Furthermore, scenarios have been described where prolonged hyperperfusion combined with pathologies such as poor vascular health (compounded by stroke pathology) can locally promote adverse events such as BBB breakdown [71, 89, 90]. Vascular health, cerebral autoregulation, and blood pressure are only several key components of an interconnected cardiovascular system that interact and drive cerebral perfusion in health and disease. Due to this complexity, the fundamental question whether and how regional cerebral perfusion should be managed is not easily answered and requires an integrative view of the entire system, which is beyond the scope of this review. Several of these integrative perspectives of cerebral autoregulation [91] and integrative CBF regulation [92] in AIS have recently been published, to which the reader is referred.

Although our understanding of PRPD has improved over the years, many fundamental questions still need to be answered. Alignment of clinical and preclinical research through methodological improvements can accelerate this process. To that end, we have identified several issues that must be addressed in both fields.

First, specific features of PRPD, such as time of occurrence, could be relevant to outcome [48]. Therefore, analysis of temporal (early vs. late and duration) but also spatial characteristics would be highly informative in clinical and preclinical research. However, studies have identified incidences of post-recanalization hypoperfusion ranging from 3 [26] to 25.3% [24]. The studies reporting these two extremes used a threshold based on contralateral reference values, where one was set at 40% [24], while the other was set at 15% [26]. Differences in operationalization of PRPD likely explain discrepancies in literature. Just as widespread adoption of standardized hypoperfusion thresholds has improved patient selection for clinical trials, so will harmonized definitions of hyperperfusion be essential for better understanding and management of this phenomenon. Initiatives such as consensus papers that outline methodology, analysis, and reporting can be a useful starting point.

Prospective (multi-center) clinical studies of PRPD are urgently needed. Retrospective studies presented here exhibit heterogeneity in methodology and patient population. Combined with small sample sizes, this can easily lead to contradictory or inconclusive results (Supplementary Table 1). It was noted that studies included in this review used various ranges of mTICI scores to classify recanalization; some used a different recanalization scale altogether, and some studies did not classify recanalization at all (in part because recanalization scales were not conventional yet). As mentioned in the introduction, hypoperfusion may be a result of failed recanalization, whereas hyperperfusion was shown to be associated with complete recanalization [25, 41, 43, 46, 93]. Hyperperfusion of certain spatiotemporal characteristics — without complete recanalization — may represent another (patho)mechanism. For unbiased study of PRPD, it is integral that either complete recanalization or the opposite is ascertained by standardized recanalization scales, which could be achieved using the mTICI [93]. Stratified analyses will continue to be essential for future prospective and retrospective analyses, especially when retrospective patient data will be pooled. Characteristics of the patient sample and reasons for exclusion, such as HT incidence and onset-to-treatment time (OTT), should be thoroughly reported. Critically, since PRPD may depend on reperfusion strategy, patients with different treatment or divergent OTT should be stratified, and perfusion data at different imaging time points should be pooled and reported accordingly. Imaging time points should be carefully chosen with as little inter-subject variation as possible.

Clinical trials should include multiparametric imaging to gain insights in phenomena associated with perfusion deficit, such as cellular damage, BBB disruption, and HT. Data on BBB disruption and HT can relay pivotal information about post-stroke lesion evolution [41]. Such extensive measurements may be challenging to perform in the hyperacute phase, yet they contribute invaluable insight into the early stages of the disease that may predict final outcomes. Conversely, preclinical studies often employ tissue status as primary outcome measures yet neglect functional outcome measures. For example, discrepancy between imaging- and behavioral outcomes [64, 66] illustrates that tissue status may not always imply functional deficiency. Simple and standardized functional outcome measures, such as a neurological deficit score [94], may be included in order to improve translation of preclinical findings to the clinic.

Notably, there has been some variability in preclinical studies regarding their success in eliciting PRPD, which may be explained by several experimental factors. As mentioned in the “Preclinical Findings of PRPD” section, choice of species and occlusion time are relevant to experimental outcome but so is the choice and execution of a particular ischemia–reperfusion model. For example, tMCAO induced by mechanical occlusion enables fast recanalization, while thrombolytic recanalization in embolic stroke models is gradual. Therefore, each model uniquely recapitulates certain treatment aspects that affect reperfusion course. In addition, animal ischemic stroke models have well-known shortcomings that can produce experimental artifacts [95, 96]. Animals are usually anesthetized during surgery and neuroimaging acquisitions, while anesthetics can differentially affect CBF [97]. Certain anesthetics, such as isoflurane and ketamine, act as vasodilators and have been shown to have neuroprotective effects [98, 99]. Taken together, these experimental factors influence study outcome and should therefore be reported carefully and consistently.

Finally, our scoping review has several limitations. Although we intended to provide a complete overview of the available literature in a reproducible way, the sheer breadth and variety of methodology and terminology used to investigate PRPD may have led to exclusion of some articles that were not covered by our search terms. In addition, queries were limited to the most used literature database, a limitation we attempted to mitigate by screening article bibliographies.

## Conclusion

In the age of reperfusion therapy, (translational) neuroimaging has the potential to elucidate causes and consequences of futile recanalization. PRPDs have been identified and are expected to play a major role in AIS outcome, yet much of their etiology remains unknown. From the results in this scoping review, it can be concluded that hypoperfusion after recanalization is undesirable. However, methods need to be developed that allow more extensive disentanglement of the underlying cause of persisting hypoperfusion, so appropriate countermeasures can be identified. Interestingly, post-recanalization hyperperfusion, which has been associated with both favorable and unfavorable outcome, seems to be more commonly reported than hypoperfusion. We summarized and interpreted evidence of the variable prognostic value of hyperperfusion, identified fundamental questions that remain unanswered and presented several methodological considerations for future clinical and preclinical studies. Together, clinical and preclinical settings should aim for a more concentrated effort in reporting and methodology, in order to reduce heterogeneity of results and enhance translational potential of findings.

### Supplementary Information

Below is the link to the electronic supplementary material.

Supplementary file1 (PDF 193 KB)
