# Supplementary material for: Implications of Post-recanalization Perfusion Deficit After Acute Ischemic Stroke: a Scoping Review of Clinical and Preclinical Imaging Studies
Source: Transl Stroke Res. 2023 Jan 19;15(1):179–94. doi: 10.1007/s12975-022-01120-6 (PMC10796479; doi:10.1007/s12975-022-01120-6)
Supplement: Supplementary file 1 — Supplementary file1 (PDF 193 KB) [file 12975_2022_1120_MOESM1_ESM.pdf]

Supplementary Table 1: Study-specific reperfusion pattern and outcome in AIS patients on time points after stroke onset.

Study (subgroup level if applicable)	N	Treatment	Perfusion imaging method	Primary outcome measure		5-18h	<12h	12-24h	24h	24-36h	48h	3d	4d	7d	9d	1m
**SPONTANEOUS REPERFUSION AND NEUTRAL OUTCOME**																
Liu et al. [84]	27	None	DSC-MRI	HT, BBB leakage			Norm			Norm	Norm	Hyper	Hyper	Hyper	Hyper	Norm
**SPONTANEOUS REPERFUSION AND FAVORABLE OUTCOME**																
Marchal et al. [9]	18	None	PET	2m MCASS and EI		Hyper										
Marchal et al. [14]	30	None	PET	2m MCASS and RI		Hyper										Norm
Crisi et al. [44]	47	None	ASL	6m NIHSS						Hyper						
**TREATMENT AND NO (INTERPRETABLE) RELATION TO OUTCOME**																
Ter Schiphorst et al. [26]	33	EVT	ASL	3m mRS						Hypo						
Brugnara et al. [93]	47	EVT	CTP	None		Hyper										
**TREATMENT AND NEUTRAL OUTCOME**																
Kidwell et al. [47]	12	IVT	DSC-MRI	3m mRS		Hyper								Hyper		
**TREATMENT AND FAVORABLE OUTCOME**																
Viallon et al. [45]	41	Antiplatelet or IVT	ASL and DSC-MRI	mRS							Hyper					
Bivard et al. [46]	100	IVT	ASL	3m mRS					Hyper							
Bivard et al. [39]	77	IVT	ASL	3m mRS					Hyper							
Yu et al. [48]	221	Miscellaneous	ASL	HT			Hyper									
Bhaskar et al. [43]	119	IVT	ASL	3m mRS				Hyper								
Lu et al. [40]	54	EVT	ASL	3m mRS									Hyper			
Potreck et al. [41]	38	EVT or EVT and IVT	DSC-MRI	3m mRS					Hyper							
Rosso et al. [25] (H/H & H/N profile)	226	IVT	ASL	3m mRS					Hyper							
**TREATMENT AND UNFAVORABLE OUTCOME**																
Yu et al. [48]	221	Miscellaneous	ASL	HT				Hyper								
Okazaki et al. [82]	31	IVT or EVT, or both	ASL	HT								Hyper				

Kosior et al. 2017 [49]	50	EVT	DSAP	HT						Hyper						
Shimonaga et al. [42]	27	EVT	ASL	3m mRS						Hyper						
Rubiera et al. [32]	151	EVT	CTP	3m mRS		Hypo										
Ng et al. [24]	130	IVT or IVT and EVT	DSC-MRI	3m mRS					Hypo							
Rosso et al. [25] (h/h profile)	226	IVT	ASL	3m mRS					Hypo							

Hyper indicates hyperperfusion; Norm, normoperfusion; Hypo, hypoperfusion; H/H, hyperperfusion in- and outside the lesion; H/N, hyperperfusion inside and normoperfusion outside the lesion; h/h: hypoperfusion in- and outside the lesion; IVT, intravenous thrombolysis; EVT, endovascular thrombectomy; PET, positron emission tomography; ASL, arterial spin labeling; DSC-MRI, dynamic susceptibility contrast-enhanced magnetic resonance imaging; DSAP, Digital Subtraction Angiography Perfusion; CTP, computed tomography perfusion; MCASS, middle cerebral artery stroke scale; EI, Martinez-Vila's evolution indices; RI, recovery index; mRS, modified Rankin Scale; NIHSS, National Institutes of Health Stroke Scale; HT, hemorrhagic transformation incidence; BBB, blood-brain barrier.

Supplementary Table 2: Study-specific reperfusion pattern and outcome in AIS animal models on time points after occlusion.

Study (subgroup level if applicable)	Animal	N	Occlusion duration (min)	Perfusion imaging method	Region of interest	Outcome measure		0-15 min	30 min	1h	1-2h	2-3h	3-6h	12h	1d	2d	3d	4d	5d	6d	7d	14d	21d	28d
**NO OUTCOME REPORTED**																								
Van Dorsten et al. [54]	Mouse	6	60	ASL	Anatomically driven ROIs	NA			Hypo	Hypo	Hypo				Hypo									
Li et al. [67]	Rat	8	30	DSC-MRI (CBF)	Anatomically driven ROIs	NA		Norm	Norm	Norm	Norm			Norm	Norm	Norm	Norm							
Van Dorsten et al. [55]	Rat	20	60	ASL	Various ADC-derived thresholds	NA		Norm	Norm	Hypo	Hypo	Hypo	Norm											
Van Dorsten et al. [55]	Rat		90	ASL	Various ADC-derived thresholds	NA		Hyper	Norm	Hypo	Norm	Norm	Norm											
Lin et al. [61]	Rat	108 (k=14)	60	ASL and DSC-MRI (CBF and CBV)	Anatomically driven ROIs	NA							Norm		Hyper		Hyper		Hyper		Hyper	Hyper		
Tanaka et al. [65]	Rat	6	60	ASL and DSC-MRI (CBF)	Anatomically and CBF driven ROIs	NA					Hyper					Hyper								
Martín et al. [59]	Rat	52	120	PET and SPECT	MCA irrigation territory	NA									Hypo	Norm		Hyper			Hyper			
Martín et al. [60]	Rat	7	120	PET	MCA irrigation territory and anatomically driven ROIs	NA									Hypo		Norm				Hyper	Norm	Norm	Norm
**NEUTRAL OUTCOME**																								
Wegener et al. [64] (reperfusion pattern A)	Rat	8	60	ASL	PWI-derived lesion	Neurological score									Hypo / Norm			Hyper				Hyper		
Wegener et al. [64] (reperfusion pattern B)	Rat		60	ASL	PWI-derived lesion	Neurological score									Hyper			Hyper				Hyper / Norm		

Wegener et al. [64] (reperfusion pattern C)	Rat		60	ASL	PWI-derived lesion	Neurological score									Hyper			Hyper / Norm				Hyper / Norm		
**FAVORABLE OUTCOME**																								
Quast et al. [56] (normo-glycemic group)	Rat	45	120	DSC-MRI (CBF)	Anatomically driven ROIs	Lesion size					Norm													
Heiss et al. [62]	Cat	17	30	PET (rCBF)	CMRglc derived threshold	Lesion size, survival		Hyper				Hypo / Norm	Hypo / Norm		Norm									
Kastrup et al. [71] (group B)	Rat	16	60	DSC-MRI (rCBF and rCBV)	Anatomically driven ROIs	Lesion size and BBB leakage		Norm			Norm	Norm	Norm											
Li et al. [66]	Rat	14	30	DSC-MRI (CBF)	Anatomically driven ROIs	Regional tissue injury, neurological score		Norm	Norm			Norm	Norm	Norm										
Lee et al. [100]	Cat	18	60	DSC-MRI (rCBV)	DWI and staining derived lesion	Lesion size				Hyper		Hyper	Norm		Norm									
Bardutzky et al. [52]	Rat	28	35	ASL	Recovered pixels (core and mismatch tissue)	Lesion size		Hypo	Hypo	Hypo	Hypo	Hypo			Hyper									
Bardutzky et al. [52]	Rat		95	ASL	Recovered pixels (core and mismatch tissue)	Lesion size		Hypo / Norm	Hypo / Norm		Hypo / Norm													
Shen et al. [63]	Rat	37	30	DSC-MRI (CBF)	Perfusion-diffusion mismatch tissue	Lesion size		Norm	Norm	Norm	Norm	Norm		Norm	Norm	Norm	Norm							
Kang et al. [57] (Wistar-Kyoto wildtype)	Rat	12	60	ASL	PWI-derived lesion	Lesion size, neurological score				Norm					Norm			Norm			Norm			
**UNFAVORABLE OUTCOME**																								
Quast et al. [56] (hyper-glycemic group)	Rat	45	120	DSC-MRI (CBF)	Anatomically driven ROIs	Lesion size					Hypo													

Heiss et al. [62]	Cat	17	60	PET (rCBF)	CMRglc derived threshold	Lesion size, survival		Hyper				Hyper / Norm	Norm		Norm									
Kastrup et al. [71] (group C)	Rat	16	180	DSC-MRI (rCBF and rCBV)	Anatomically driven ROIs	Lesion size, BBB leakage		Hyper			Norm	Norm												
Takamatsu et al. [68]	Cyno-molgus monke y	13	180	PET	Anatomically driven ROIs	Regional tissue injury		Hyper				Hypo												
Lee et al. [100]	Cat	18	60	DSC-MRI (rCBV)	DWI and staining derived lesion	Lesion size				Hyper		Hyper	Norm		Hypo									
Wang et al. [70]	Rat	20	80	ASL	Anatomically driven	Regional tissue injury			Hypo		Norm	Norm	Norm		Norm	Hyper	Hyper	Hyper	Norm	Hyper				
Lee et al. (pattern I) [69]	Cat	10	60	DSC-MRI (rCBV)	Anatomically driven ROIs	Regional tissue injury				Hyper					Hyper / Norm	Hyper / Norm								
Lee et al. (pattern II) [69]	Cat		60	DSC-MRI (rCBV)	Anatomically driven ROIs	Regional tissue injury				Hyper					Hypo	Hypo								
Bardutzky et al. [52]	Rat	28	35	ASL	Non-recovery pixels (core and mismatch)	Lesion size		Hypo	Hypo	Hypo	Hypo				Hyper									
Bardutzky et al. [52]	Rat		95	ASL	Non-recovery pixels (core and mismatch)	Lesion size		Hypo	Hypo		Hypo													
Shen et al. [63]	Rat	37	30	DSC-MRI (CBF)	Core tissue	Lesion size		Norm	Norm	Norm	Norm	Norm		Norm	Hyper	Hyper	Norm							
Kang et al. [57] (spontaneous hypertensive)	Rat	12	60	ASL	PWI-derived lesion	Lesion size, neurological score				Hypo					Hypo			Hypo / norm			Hypo / norm			
Burrows et al. [53]	Mouse	15 (9)	30	2D optical imaging SPECT	Somato-sensory cortex	Regional tissue injury		Norm					Hypo											
Tiwari et al. [72]	Rat	12	60	ASL	ADC/PWI-derived lesion	BBB leakage		Norm								Hyper								

PET indicates positron emission tomography; SPECT, single-photon emission computed tomography; DSC-MRI, dynamic susceptibility contrast-enhanced magnetic resonance imaging; ASL, arterial spin labeling; ADC, apparent diffusion coefficient; PWI, perfusion-weighted imaging; BBB, blood-brain barrier; TUNEL, TdT-mediated dUTP-biotin nick end labeling; CMRglc, cerebral metabolic rate of glucose; Hypo, hypoperfusion; Norm, normoperfusion; and Hyper, hyperperfusion.
